# Policies Make Coherent Care Pathways a Personal Responsibility for Clinicians: A Discourse Analysis of Policy Documents about Coordinators in Hospitals

**DOI:** 10.5334/ijic.3617

**Published:** 2018-07-10

**Authors:** Audhild Høyem, Deede Gammon, Gro Rosvold Berntsen, Aslak Steinsbekk

**Affiliations:** 1 Centre for Quality Improvement and Development, University Hospital of North Norway, Box 20, N-9038, Tromsø, NO; 2 Norwegian Centre for E-health Research, University Hospital of North Norway, Box 35, N-9038 Tromsø, NO; 3 Center for Shared Decision-Making and Collaborative Care Research, Oslo University Hospital HF Division of Medicine, Box 4950 Nydalen, N-0424 Oslo, NO; 4 Department of primary care, Institute of Community medicine, UiT The Arctic University of Norway, Tromsø, NO; 5 Department of Public Health and Nursing, Norwegian University of Science and Technology, Box 8905, N-7491 Trondheim, NO

**Keywords:** care pathway, coordinated care, continuity of care, complexity, integrated care, hospitals

## Abstract

**Introduction:**

In response to increase of patients with complex conditions, policies prescribe measures for improving continuity of care. This study investigates policies introducing coordinator roles in Norwegian hospitals that have proven challenging to implement.

**Methods:**

This qualitative study of policy documents employed a discourse analysis inspired by Carol Bacchi’s ‘What‘s the problem represented to be?’. We analysed six legal documents (2011–2016) and selected parts of four whitepapers presenting the statutory patient care coordinator and contact physician roles in hospitals.

**Results:**

The ‘problem’ represented in the policies is lack of coherent pathways and lack of stable responsible professionals. Extended personal responsibility for clinical personnel as coordinators is the prescribed solution. Their duties are described in terms of ideals for coherent pathways across conditions and contexts. System measures to support and orchestrate the individual patient’s pathway (e.g. resources, infrastructure) are scarcely addressed.

**Conclusions and Discussion:**

We suggest that the policies’ construction of the ‘problem’ as a responsibility issue, result in that neither diversity of patients’ coordination needs, nor heterogeneity of hospital contexts regarding necessary system support for coordinators, is set on the agenda. Adoption of rhetoric from diagnosis-specific standardized pathways obscures unique challenges in creating coherent pathways for patients with complex needs.

## Introduction

The number of people with chronic conditions and multiple health problems are growing [1,2]. Common to these conditions, are the person’s need for access to integrated healthcare from a range of services and professions over time to ensure continuity of care [3]. Modern healthcare organizations are responding to this need by organizing the services according to a process-perspective, as opposed to an episodic focus in care delivery [4,5]. While a multitude of concepts, definitions and models are in use [6], the aim of continuity of care is frequently expressed as coherent care pathways [4,5,7,8,9].

Similar to several Western countries [10,11,12,13,14,15], Norway has implemented national reforms aiming to overcome challenges due to fragmentation of services for persons with long-term healthcare needs. The Norwegian Coordination reform was introduced in 2008 and implemented in 2012 [16]. Central measures are transfer of tasks and responsibility from hospitals to primary healthcare, mandatory collaboration contracts between hospitals and municipalities, establishing new service units for more advanced treatment in primary healthcare, as well as the introduction of penalty fees for primary healthcare when they delay in receiving patients ready for discharge from hospitals [16]. These policies have implications for hospitals’ roles and responsibilities; such as shorter hospital stays, restrictions to the most specialized treatment and care, as well as the need for enhanced collaboration and coordination towards primary healthcare [3,17,18,19].

In addition to coordination measures on the administrative level, the Norwegian healthcare acts were amended from 2012 with new patient rights as well as statutory obligations to appoint patient coordinators on the operational level both in primary and specialized healthcare [20,21,22]. The hospitals became legally obliged to assign patient care coordinators to patients with complex or long-term needs of care [23]. Moreover, a new role for ‘contact physician’ came into force in 2016, both as a patient right and as a legal duty for the hospitals [24]. This responsibility applies to patients with serious conditions [20]. The authors had no relation or commitment with the policy development.

These new coordinator roles represented a reinforcement of a long-term initiative: Since 2001, Norwegian health authorities have focused on individual care plans and patient coordinator roles held by clinicians to secure individualized and integrated care, with gradually stronger legal regulations [11,20]. Research prior to the Coordination Reform has shown that implementation of patient coordinator roles for complex needs had been slow, that professionals were reluctant to take on such roles, and that the proportion of individual care plans relative to the estimated number of persons who qualify for such plans was low [11,17,25,26]. The Auditor General of Norway concluded in 2016 that, despite the extensive legislative efforts, the coordinator roles and individual care plans did not secure cohesive pathways for persons with complex or long-term needs according to the policy intentions [27]. Attempts to improve coordination at the operative level through legislation, has showed limited results also in other European countries [12,28].

The aim of this study was to explore discursive aspects of Norwegian policy documents that legislate two coordinator roles in hospitals to ensure coherent care pathways for patients with complex or long-term healthcare needs. Our analysis was guided by sociologist Carol Bacchi’s analytic framework, ‘What‘s the problem represented to be?’ (WPR) [29]. By examining how the ‘problem’ to be solved by these coordinator roles was constructed in the policy documents, we offer a critical reflection on the substantive content of this policy initiative [30].

## Methods and materials

### Design

Bacchi’s approach builds on that every policy proposal contains an explicit or implicit diagnosis of the ‘problem’ that the policy aims to solve. In the WPR-perspective, the ‘problems’ are not objectively given; rather they are constructed as part of the policymaking process [29,30]. In accordance with Bacchi, we use quotation marks around the word ‘problem’ when it refers to the kind of change implied in a particular policy proposal and is not used in the traditional meaning [29]. The WPR-analysis is directed towards making the implicit ‘problems’ explicit, and to scrutinize them closely. In line with the aim of our study, we apply four of the six analytic questions posed by Bacchi to guide the analysis [29]. The WPR-analysis starts with ‘working backwards’ from the proposed policy intervention to make explicit what is defined as in need of change (Question 1). We then proceed with scrutinizing the conceptual premises or discourses that enables particular promises and policies to be developed (Question 2). The next step is to identify and reflect upon what is left silent or unaddressed and thus is not made subject to policy goals or measures (Question 4). Finally, the implications for the roles or positions of those affected by the current ‘problem representation’ are in focus (Question 5). Bacchi’s remaining questions (3 and 6), that deal with how the ‘problem representation’ has come about and how it has been disseminated and defended, are not addressed in this study.

### Historical background and context for the studied coordinator roles

Norway has a publicly funded healthcare system mainly free of charge for the citizens. Primary healthcare is organized by the municipalities and comprises of home care and nursing services, nursing homes, physiotherapy, occupational therapy etc. Every citizen has the right to a personal family doctor. The family doctors are gatekeepers for specialized healthcare and have a coordinating role for the totality of medical healthcare for the patient [31]. Most family doctors are organized as private enterprises, with designated public responsibilities [17]. Specialized healthcare is run and owned by national health authorities, and is organized into four regional health enterprises that manage 20 hospital trusts [17].

From 2001, individual care plans were introduced as a statutory right for those with complex care needs [32], and as a duty for healthcare personnel both in hospitals and primary care (Table 1). Individual care plan is a personally tailored plan built around prioritized personal goals for the patient, covering needed services from different sectors and units. It is central that the plan is developed in a partnership between the patient and a multidisciplinary team of professionals from relevant service units, led by an individual care plan coordinator from the clinical staff. It is the duty of the plan coordinator to recruit and organize the participation of relevant professionals for this work [17]. At the same time, Norwegian hospitals were obliged to appoint a ‘patient responsible physician’ for all hospital patients [33]. As previously mentioned, several amendments were made in the healthcare acts and regulations as part of the Coordination reform in 2012 [22]. The patient care coordinator for patients with complex or long-term needs of care became a freestanding role, regardless of if the patient needed or wanted an individual care plan [23]. This modified coordinator role replaced the ‘patient responsible physician’.

**Table 1:** Historical development of coordinator roles in Norwegian hospitals 2001–2016.

Coordinator roles in hospitals	Year of introduction or change			

	2001	2012	2015	2016

Individual care plan that includes a personal coordinator for patients with long-term complex needs. (Patient right and healthcare obligation by law, 2001)	X	X	X	X→
Patient responsible physician. All patients. (Regulations 2001)	X			
Patient care coordinator in specialized healthcare for patients with long-term complex needs, whether they want an individual care plan or not. Preferably a physician. (Healthcare obligation by law 2012–2015)		X		
Coordination unit in each hospital. Responsible for the hospital’s work with individual care plans and coordinators. (Regulated since 2001, obligation by law in 2012)	X	X	X	X→
* Patient care coordinator in specialized healthcare. The coordinator may have any health profession. (Law amendment 2015)			X	X→
* Hospital contact-physician for seriously ill. (Patient right and healthcare obligation by law 2016)				X→

* These two roles are the focus of this study.

A parallel process in Norwegian hospitals, also focusing on pathway coordinator roles, was the development and implementation of standardized clinical pathways for 28 types of cancer in 2015 [34]. A clinical pathway describes a process within a hospital or clinic, whereas a care pathway includes discharge, follow up and out-patient clinic activities [9]. The cancer pathways are designed according to international guidelines that are customized to the local contexts. It is mandatory that each pathway have a cancer pathway coordinator in a designated position to guide the patients through the programme, to monitor and register that the events follow the plan, as well as to participate in multidisciplinary work and take care of logistics. The cancer pathway coordinator role is not decreed by law. The national implementation support to the standardized cancer pathways has been extensive [34].

### Materials

In accordance with Bacchi [29] we chose the law paragraphs in the Specialized Health Services Act covering the two legally obligated coordination roles as an entry to the field [20]. The included documents are presented in Table 2, and is referred to in the result section by the document numbers in the table (in round brackets). Further, we included all the legal documents concerning the responsibility of specialized healthcare related to these roles: The regulations (2) and directives (3) applicable for the chosen law paragraphs, the government law-proposition that introduced the amendment concerning the contact physician (4), and the national guideline that includes the patient care coordinator role (5). Finally, we included the national guideline for implementing the contact physician that was published during the process of the study (6). From the documents that comprised of more than the studied roles, paragraphs and sections covering the chosen coordinator roles were selected (specified in Table 2). We have excluded parliamentary proceedings and media communications. To provide context and historical background, four whitepapers (7–10) that introduce, justify or refer to the studied roles and their predecessors were included.

**Table 2:** The included documents.

Document number, document title and which parts of the documents are analysed		Type and status	Topic covered*	Publication year

1.	Specialized Health Services Act [20]. §§ 2–2, 2–5a, b and c	Current legislation	PCC & CP	1999, updated 17.6.2016
2.	Regulations to the Specialized Health Services Act and the Health and Care Services Act concerning rehabilitation, individual plan and patient care coordinator [35].	Regulations covering the patient care coordinator role	PCC	2012
3.	Directive to the Specialized Health Services Act [23]. p. 23–27	Circular	PCC	2013
4.	Law proposition to the Parliament, Prop. 125 L. Amendments to the Specialized Health Care Act. [22]. Chapters 1–8, p. 5–38 and 10, p. 43–46	Proposition The proposed amendment to the Specialized Health Services Act was approved in November 2015	PCC & CP	2014–2015
5.	Guidelines for patient care coordinator [36]. Chapter 13, p. 82–93	Document with recommendations and clarifications for how to understand the law paragraphs and regulations regarding rehabilitation, individual care plan and coordinator	PCC	2015, updated 23.2.2017
6.	Guidelines for contact physician [37]. Chapters 1–8, p. 1–33	Document with recommendations and clarifications for how to understand the law paragraphs and regulations regarding contact physician in specialized healthcare	PCC & CP	2016
7.	The Coordination Reform. Proper treatment – at the right place and right time. Report No. 47 (2008–2009) to the Storting. [16]. Chapters 1–5, p. 11–53 and 10, p. 111–114	Report to the Storting from the Minister of Health and Care Services	Background and context	2009
8.	NOU 2005: 3. From piecemeal to whole – an integrated health service [38]. Chapters 1, 2, p. 11–21, 4, p. 40–48, 6 and 7, p. 67–87	Official Norwegian Report delivered to the Ministry of Health and Care Services	Historical background	2005
9.	NOU 1997: 2 The patient first! Leadership and organization in hospitals [39]. Chapters 2, p. 15–16 and 8, p. 92–108	Official Norwegian Report delivered to the Ministry of Social Affairs and Health	Historical background	1997
10.	Meld.St.11 (2015–2016) National health- and hospital plan 2016–2019. [40]. Chapter 7.3, p. 57–58	Report to the Storting from the Minister of Health and Care Services	Current plan for hospitals	2015

* The abbreviation PCC is used for patient care coordinator and CP for contact physician in table 2.

### Analysis

We started with mapping central characteristics of the two coordinator roles based on full text readings of the selected documents and document sections. AH consecutively entered condensed descriptions of the roles in a table (Microsoft Excel) according to dimensions that were inductively developed.

The next step in the analysis of the selected documents and document parts were conducted within NVivo, a computer program for qualitative data analysis [41]. The following terms were identified as central in the characteristics of the coordinator roles in the aforementioned mapping process: coordinator, contact-physician, continuity, coherence and compound words containing the Norwegian word ‘forløp’ (English: pathway, trajectory, course, path). Automatic text searches were conducted in NVivo for these terms. This was done in order not to miss any of the text covering central concepts describing the roles, tasks and aims.

At this point, two of Bacchi’s guiding questions for the analytic process [29, p. 2] were applied: 1. What’s the problem represented to be? 2. What presuppositions or assumptions underlie this representation of the ‘problem’? First, the full text was read in the light of these questions. Subsequently, the result was validated through reading of the central paragraphs that had been identified through the text searches in NVivo. ‘Answers’ to each question found in the texts were recorded successively in memos. Further, text excerpts representing central themes were included.

Memos covering analytic reflections were written during the process of coding and in discussions with the co-authors when themes began to take shape that were relevant for the analysis. Then the memos were read together with the full texts and the coded texts, and an analytic matrix was made in a spreadsheet. The themes were gradually condensed and abstracted both within and across the studied coordinator roles. The findings were then categorized into the two themes presented in the second part of the result section; “What is the problem represented to be?”.

Finally, the fourth and fifth of Bacchi’s questions were employed as a basis for the discussion section; 4. What is left silenced? 5. Which effects are produced by this ‘problem representation’?

A log was kept to document the analytical steps taken during the process.

## Results

### Characteristics of the coordinator roles

Table 3 presents an overview of central characteristics for the patient care coordinator and the contact physician respectively: purpose, tasks, assigned profession and target group, as well as legal status and implementation status.

**Table 3:** Central characteristics of the two coordinator roles.

Area	Coordinator	

	Patient care coordinator (1,2,3,4,5)	Contact physician (1,4,6)

Purpose	Ensure continuity and coherence in patients’ care pathways.	Enhance the quality of treatment. Contribute to patient safety, predictability and continuity in patients’ pathways.
Tasks	Follow up of the individual patient before, under and after hospital stay. Coordinate hospital services between units, departments, and professionals around the patient. Be the point of contact for the patient, collaborating professionals, external service providers and institutions. Secure information and dialogue with the patient. Contribute to progression in the process in the work on the individual care plan (ICP) when this is applicable*	Be a stable contact-person for the patient regarding medical questions. Be involved in treatment or follow up, and be available and inform the patient and next of kin through the course of treatment and follow up. Contribute that the patient trajectory develops as planned. Establish contact with other professionals/units if necessary. Be available for medical questions from primary healthcare or other professionals. The hospital can decide whether the contact physicians also should hold the statutory responsibilities for ‘information to the patient’ and ‘documentation in the patient record’.
Assigned profession	Healthcare personnel. (From 2012–2015: ‘Coordinator should preferably be a physician’. This was removed in 2015 in an amendment of the law paragraph).	Physician with relevant competence, preferably a specialist. In mental healthcare and substance abuse treatment, contact psychologist may be appointed in place of contact physician.
Target group	Patients with complex or long-term needs of coordinated services under the Act of specialized healthcare.	Patients with serious conditions who are in need of treatment or follow up from specialized healthcare for a period of time.
Criteria defining target group	Expected needs of services for the patient from different departments, units and professions in specialized healthcare over time, and the need of coordinated services.	The severity of the condition; risk of disability or death, comorbidity, expected progression. Duration: Need of treatment more than 3–4 days. Need of more than one follow-up consultation.
Legal status	Obligation for specialized healthcare (Specialized Health Services Act). Not a legalized right for the patient.	Obligation for specialized healthcare (Specialized Health Services Act). Legalized right for the patient (The Patients’ Rights Act).
Implementation status	Various degree of implementation and knowledge in the hospitals (4). National Audit concludes that the goals are not achieved [27].	Act came into force September 2016. The hospitals are in the process of developing routines for the role as well as procedures and tools for documentation and communication (2017).

* From being a common responsibility for all healthcare services, the main responsibility for individual care plans was assigned to the municipalities from 2012. When patients need services from both primary and specialized healthcare, the hospitals’ responsibility was confined to informing the patients, reporting patients’ needs of individual care plans to the municipalities, and to collaborate and contribute according to the needs of the individual patient. Specialized healthcare must develop the plan together with the patient, if he or she do not need services from the municipality (5).

### ‘What is the problem represented to be?’

We identified the following overarching ‘problem’ that the introduction of statutory patient care coordinator and contact physician roles in hospitals are designed to solve:

The hospitals cannot be trusted in providing coherent care pathways, nor do they ensure responsible and available clinicians for patients with complex long-term needs of care.

The central aspects of this ‘problem representation’ were categorized into two themes: 1. ‘Lack of pathway-organized services’ and 2. ‘Lack of stable and responsible clinicians. Separating the desired service-organization (theme 1) from the responsibility for providing it (theme 2), helped highlight important and contrasting topics. However, the themes are closely related and thus overlap somewhat in the following presentation.

#### Lack of pathway-organized services

The central concepts used in the descriptions of the two coordinators’ roles in the policy documents express ideals of continuity and holism, and that healthcare is planned and delivered as a process around the individual patient in the form of a coordinated care pathway or trajectory.

“The coordinator must accommodate the patients’ needs for a continuous and holistic patient pathway” (3, p. 25).

The arguments in policy documents for establishing the coordinator roles show the type of ‘problems’ that the coordinators should help alleviate.

“Hospitals are complex organisations. Patients move between outpatient and inpatient services, x-rays, labs, and different clinical departments, and between hospital and primary health and care services. It is well documented that the risk for failure is greatest during transitions between services. Many patients complain about fragmented pathways, many different clinicians involved, poor information flow, and lack of continuity and overview” (10, p. 57).

We see similar descriptions of ideals and challenges in the other documents (4, 5, 6, and 8). The key terms used depict a planned process along a timeline. They include; ‘pathway’, ‘patient pathway’, ‘cohesive pathway’, ‘standard pathway’, ‘course of treatment’, ‘patient trajectory’ and similar. These terms are used in conjunction with words as continuous, holistic, coherent and coordinated. Clear definitions of these concepts are not offered. To describe how the different key terms are applied in the documents, they are categorised in three different dimensions of care; a safety and quality dimension, a temporal and organizational dimension, and a patient involvement dimension.

##### The dimension of safety and quality in care

The regulations of these coordinator roles state the ideal that the patients at all times must feel safe and secure in their encounters with healthcare.

“It is vital that patients with a serious illness, injury or disability, and also their next of kin, feel secure throughout the patient trajectory” (4, p. 10).

The coordinators are ascribed a role of monitoring and adjusting the ongoing service provision to safeguard quality and patient safety for the individual patient.

“The aim [of contact physician] is increased security and predictability for the patient and next of kin, improved quality and cohesion in the medical services” (10, p. 57).

The documents do not outline further how to interpret safety and quality. Nor are the tasks and magnitude of the responsibilities for the coordinators described when it comes to ensuring each individual patient’s experience of security, overview, predictability and coherence at any stage in the healthcare trajectory.

##### The temporal and organizational dimension of care

The descriptions of the coordinator roles underline the importance of seeing the elements of healthcare in a broader holistic perspective, or as a phase in a longer trajectory (temporal dimension), where attention to transfers is vital in preventing fragmented care (organizational dimension) (5).

“Achieving a good pathway requires good collaboration, logistics and communication between the various service localities and levels of care” (4, p. 10).

In this perspective, the ideals of continuity and holism imply services delivered in a timely fashion according to a plan, along a timeline adapted for the individual patient. It is specified that the coordinator shall ensure coordination of all the healthcare services that are relevant in connection to a hospital stay (3). In the guidelines (5, 6) it is referred to the standardized pathways that in 2015 were implemented in Norwegian hospitals for 28 cancer diagnoses [34], and that the cancer pathway coordinators may fill the role as ‘patient care coordinators’.

##### The dimension of patient involvement in care

The coordinators are expected to ensure that the process of planning and performance of care fulfils specific ideals of person-centred care. One aspect is to ensure user participation and patient influence through co-creation of care (5).

“The coordinator is responsible for following up input from patients and their families, and for ensuring user involvement and good dialogue” (3, p. 25).

The law paragraph (1, §2–5 a) states that the patient care coordinator shall safeguard progress in developing and following up the patient’s individual care plan when applicable. The reference to the statutory individual care plan signals specific requirements for personalisation of the care planning and delivery in the form of a written plan based on personal goals. Additionally, it signals that the scope of what is within the coordinators responsibility is the patient’s ‘coping with life’, not merely treatment or healthcare.

#### Lack of stable and responsible professionals

The law requires that hospitals appoint patient coordinators from clinical staff to be responsible for ensuring continuity of care for individual patients (1, §2–5 a and §2–5 c). This implies an assumption that current fragmentation is the result of lack of effort on the part of coordinating professionals. Thus, the ‘problem’ becomes the lack of a responsible and available person.

The patient care coordinator role is described in the documents by terms such as; ‘ensure’, ‘provide’, ‘secure’, ‘safeguard’, ‘take responsibility for’, ‘take care of’, ‘at any time hold the main responsibility for’, and ‘have a central role in’. These reflect expectations that the coordinators should take on a high degree of personal responsibility. The tasks connected to these responsibilities include; ‘the needed follow-up of the individual’, ‘coordinated and individualized services’, ‘co-ordination with external services’, ‘progress in development of individual care plan’, and ‘dialogue and user participation’ – among others (1, 2, 3, 5, 6).

“The coordinator is a service provider responsible for ensuring necessary follow-up and coordination of the services, as well as progress in work with the individual care plan” (5, p. 83).

Some of the same terms are used to describe the role of the contact physician: ‘Keep the complete overview over the patient trajectory’, ‘contribute to ensuring that the trajectory develops as planned’, and ‘[it is recommended that the contact physician] hold the statutory role of being responsible for information to the patient and for the patient’s record’ (4, 6).

“Patients will still meet several physicians during the process, but patients and next of kin must feel secure that there is one physician who has a particular responsibility for them” (6, p. 5).

The way the pathway ideals and responsibilities are described, it is not clearly specified whether the pathway coordination responsibilities for these coordinators are limited to the hospital treatment, the totality of the patient’s healthcare encounters across institutions, or if it should cover a wider coping with life-perspective. Particularly the references to the individual care plans in the descriptions of the patient care coordinator, indicates that the scope of this role includes the patients’ process of ‘coping with life’ in addition to disease-related treatment and the logistics of the healthcare events both before, under and after hospital treatment (3, 5). Also the references to ‘necessary follow-up’, ‘complete overview of the patient trajectory’, and ‘coordination with external services’ express wide and undefined responsibilities.

It is recommended that the patient have only one contact physician, also when several healthcare units are involved (6). The responsibility of the contact physician is thus not limited to the treatment within the unit where the physician is organized, the areas of the physician’s specialization, or the period of hospital stay (6). Additionally, the guidelines for the contact physician explicitly state that taking on this role in no way changes the responsibilities of existing roles like ‘treatment responsible physician’ and ‘responsible surgeon’ (6, p. 6). In the same way, the responsibility of the patient care coordinator is to interact with and orchestrate personnel both within and outside the unit or institution that have or will have treatment or follow-up responsibility for the patient (5).

One of the main aims of both roles for patients with long-term, complex needs is to provide relational continuity between the patient and a particular healthcare professional who is available over time, and who is well informed about the patient’s situation and the relevant services to be coordinated (5, 6).

“The patient must experience that the contact physician represents continuity throughout the treatment pathway. (…) The contact physician must provide the patient with information, be available, and participate in the treatment team” (6, p. 21).

About the patient care coordinator it is said that:

“The service provider who is appointed as coordinator must at all times have the main responsibility for follow-up of the patient” (5, p. 83).

Patients with a combination of complex long-term needs and serious illness qualify to have both a contact physician and a patient care coordinator (1). In addition, those who have a cancer diagnosis, for which a standardized pathway has been implemented, may also have a cancer pathway coordinator (6). The tasks and responsibilities are to some extent differentiated between the roles in the current guidelines for situations where the patient qualifies for more than one of these coordinators (4, 5, 6): The care coordinator’s role should be limited to the logistical coordination and to ensure progress in individual care plan process. The contact physician’s role is mainly to be informed and available for the patient, next of kin and collaborating healthcare personnel in relation to medical issues. While the cancer pathway coordinator is recommended to fill the role of patient care coordinator if the patient needs both (5, 6). The need of close collaboration and clarification of the roles towards the patients is emphasized (6). Further distinctions between responsibilities and roles in varying circumstances are not offered in the documents, nor are questions related to capacity and authority within and between the roles addressed.

## Discussion

This study found that the policies prescribing contact physicians and patient care coordinators are designed to solve the ‘problems’ that the hospitals do not provide coherent care pathways for patients with complex long-term needs of care, and that they do not ensure responsible and available clinicians as stable contact persons for these patients. Professionals in clinical staff should be appointed as coordinators who are assigned a personal responsibility for realization of healthcare that is planned and delivered as a process around the individual patient in the form of a coordinated care pathway or trajectory. This covers quality and security, time/space logistics and processes that ensure patient involvement. The coordinators are also expected to be available for the patients, and responsible on behalf of the system, over time and across units.

### Common pathway rhetoric – different premises

The call for pathway approaches in the studied documents resonates with the central public discourse expressing the ideal of process-organized services aiming at efficient delivery, equality of healthcare and continuity of care for patients [4,5,7,8]. The many dimensions of care pathway ideals described as the coordinators’ responsibility in the documents, can be expected to represent challenges for professionals and leaders responsible for realizing these. Different understandings of the care pathway concepts may embody differing, and in some cases even contradictory logics, processes and knowledge bases [42].

Complexity is a key concept used in defining the target groups for the hospitals’ obligation to appoint patient care coordinators. Complexity is defined as the need for services from two or more units or professions over time [23]. Thus, the predictability of the care process and the needs for services and coordination assistance will show considerable variation within the target group. This will in turn have an impact on which type of care pathway model that is relevant in each case. The European Pathway Association distinguish between ‘chain models’ for high degree of predictability of needs and agreement about treatment, ‘hub models’ with a case manager for less predictable processes, and ‘web models’ for changing and unpredictable situations [7]. For the part of the target group with multimorbidity and serious illness that might be progressive or result in severe disabilities, a variety of services may become relevant at different stages for the individual patient [43]. Processes may be messy due to parallel ongoing assessment and treatment, many units and actors involved, and unpredictable disease progression or complications, or because of the patient’s personal situation [44]. Thus, there will be variation in what is needed to provide coherent pathways for the individual patient according to the policy ideals. However, the policies do not address how variation in degree of complexity of the patients’ needs may have consequences for the coordinator roles.

Additionally, the pathway rhetoric in the studied documents coincides with the rhetoric around standardized clinical pathways in the hospitals [9,45]. The Norwegian cancer pathways is an example of such pathways [34]. These are preplanned ‘chains of care’, often with pre-booked consultations along a defined timeline, organized multidisciplinary collaboration and dedicated coordinator positions with defined mandates and responsibility for facilitating, monitoring, customizing and documenting the patient flow. For this type of pathways, limited to a defined diagnosis or treatment procedure, there are clinical guidelines to be followed and a common knowledge base to build on [4]. The premises for such pathways are sufficient predictability of the patients’ needs and agreement about treatment to make a planned chain of care [7]. Moreover, the standardized pathways are limited to a particular context or institution, and the organization of these pathways builds on local workflow analyses [45]. The statutory coordinator roles, however, are universal. Thus, the coordinators are expected to realize tailored pathways based on the individual patient’s needs in hospital units and beyond with various infrastructure available.

The expansion of process-organized ideals for patient pathways from one diagnosis, to encompassing multi-morbidity or complex service needs, as described by Fineide and Ramsdal [4], were also identified in our investigation. Similarly, we found a vertical expansion of the care to be organized, from relatively short intra-organizational processes, to cover transfers between services across organizational borders over long periods. Consequently, the knowledge base may be both controversial and uncertain [4], thereby failing to fulfil a central precondition for standardizing pathways [7]. Nevertheless, we see that the same concepts are used, without problematizing these factors. The hospitals are even advised to look to the implementation of the cancer pathways [22] for how to meet the expected challenges related to implementing the contact physician role. The policy documents do not mention the extensive preparatory work, structural organization and dedicated professional resources on which the cancer pathways are based [34]. No such processes or resources are referred to for the two studied coordinator roles. Beyond creating an individual care plan, no models or methods are suggested.

Based on the above, we suggest that the use of rhetoric based on clinical pathways, obscures the particular challenges related to creating coherent care pathways for patients with long-term needs, multimorbidity, low predictability of needs, or involvement of several service units and institutions. Additionally, we argue that as a result of the ‘problem representation’ inherent in the studied policy documents, the diversity of patient needs and preferences as well as the heterogeneity of current hospital work practices are left silent. Thus, the need of structural arrangements like availability of multidisciplinary resources, organized teamwork, mandate to cross borders, or process redesign are not thematised.

### Personal versus systemic responsibility

Both the studied coordinator roles depend on assigning personal responsibility to a named and available clinician. Seemingly, this is a reasonable solution to fragmented services and low personal continuity as experienced by patients in hospitals. Having one trusted clinician, who helps navigate the system and takes personal responsibility for patient involvement and care planning, is understandably highly valued by patients with long-term healthcare needs [44,46]. Nevertheless, as others have noted [47], designating responsibility to individual professionals may threaten the collective organizational responsibility needed to ensure ‘system continuity’ on a 24/7 basis in the hospital. Krogstad et al. point out differences between visible continuity measures such as when the patient meets the same professionals every day (front stage continuity), and continuity that is taken care of behind the scenes within an organizational system securing shared information and responsibility (back stage continuity). The current ‘problem representation’ is conceptualized as lack of ‘front stage continuity’ [47], and thus the answer is that professionals extend their personal responsibility for the follow-up of individual patients.

Still, some sort of personal responsibility is not new to healthcare. Conscientious healthcare professionals engage daily in detecting and bridging gaps of care, and work to build continuity for patients [48]. Organizing work is often invisible, as when nurses create continuity for patients across shifts, departments and institutions through a proactive identification of actions that Allen call “trajectory mobilisation” [49]. This type of work demands competence and experience, and it is context sensitive [50,51].

Additionally, numerous coordinator roles are established on a system level in hospitals; some in dedicated positions, with earmarked resources and infrastructural support. These may be devoted e.g. to patient discharge [52], to the follow up of patients with a particular diagnosis [53], or to patients undergoing a certain treatment procedure [54]. However, the studied Norwegian coordination policy refer in a very limited degree to existing coordination measures, established coordinator roles, or to research on such. Thus, those responsible for implementing the new statutory coordinator roles must agree on how to interpret the policy demands, and how to design the new roles in relation to existing resources in the particular context.

### Extended and overlapping scope of responsibility

The introduction of the patient responsible physician in 2001 (Table 1), was found to disrupt established work practices and distribution of responsibility among nurses and physicians in hospitals [51]. The hospital trusts and professionals’ unions that submitted consultation responses to the proposition of the law amendment introducing contact physician in 2014 [55], expressed concern that this obligation of personalized responsibility would interfere with established roles and create new grey zones of responsibility in the hospital, thus increasing the risk of poorer security and quality of care for the patients. While sharing the ideals of process-organized healthcare around the individual patient, several considered this arrangement neither feasible nor sustainable [55].

The two studied hospital coordinators’ responsibility covers coordinating care in relation to hospital admissions, including before, between and after hospital treatments [36,37]. Hence they are expected to choose their actions and priorities, and allocate the needed resources based on an overall perspective of the patient trajectory [56], thereby extending their professional responsibility both in time and scope. This includes defining when there is a need to apply the statutory coordinator role, and negotiating the practical performance of the role in the local work practices.

This extended duty also actualizes questions related to distribution of responsibility for care coordination between specialized and primary healthcare. The family doctor in primary care has a long-standing responsibility of coordinating medical healthcare for the individual patient [31]. The new contact physician in hospitals now has a similar duty, though with the main focus on the specialized healthcare [37]. The guideline for contact physicians specify that implementing the new roles should not entail changes to current responsibilities between primary and specialized healthcare [37]. The patient care coordinators in primary healthcare are given a nearly identical duty as those in hospitals [35]. Additionally, the municipalities are assigned the main responsibility for coordination through the individual care plan when the patient needs services from both sectors [35]. The new statutory coordination responsibilities for the hospitals thus seem to overlap those of primary healthcare. Hence, the distributions of responsibility between coordinators in the different sectors are subject to negotiations in each given situation. Limited system support is available for such processes. The statutory Coordination units are responsible, on behalf of the hospital and the municipality respectively, for realization of individual care plans and patient care coordinator roles as well as for supporting the coordinators [35]. Two unpublished surveys by the first author show, however, that a majority of these units in hospitals have limited capacity and authority. Often they consist only of one part-time position, and have limited authority due to a low position in the organization. Many also describe a low degree of leadership support.

A coordinator role that implies actions beyond both professional and institutional mandates, leads to authority challenges such as restricted access to relevant arenas and information, as well as lack of decision-making authority needed in the realization of individual care pathways across units and sectors. An apparent paradox is that at the same time as responsibilities for the follow up of patients with long-term complex needs are transferred from hospitals to primary healthcare as part of the coordination reform, the hospitals’ duties for cross-sector coordination are both extended and strengthened through legal obligations.

### Limitations

To the best of our knowledge, the document sample covers the current Norwegian legislation on our topic. An inclusion of governmental debates, media communication and a wider range of governing documents could have broadened the scope and analysis.

One of the main aims of Bacchi’s post-structural discourse analysis is to disclose how the way the ‘problem’ is represented in policy, makes certain subject positions possible or available [30]. The focus of our study is limited to the subject positions of healthcare providers. The study would have been strengthened by an examination of the policies’ implications for the patient role and position, and for the fulfilment of patients’ rights.

An empirical study of stakeholders’ understanding of these policies would offer important complementary insights.

## Conclusion

Our analysis shows that the lack of coherent pathways, as well as the lack of stable and responsible professionals for patients with complex needs are represented as the ‘problems’ to be solved by extending individual healthcare professionals’ scope of responsibility in roles as coordinators. We suggest that the policies’ construction of the ‘problem’ as a responsibility issue shows that Norwegian authorities focus on ‘front stage continuity’. Professionals and leaders in hospitals emphasize ‘back stage’ or system continuity, and have criticized the policymakers for not taking into account the diversity of patient needs, the heterogeneity of hospital practices and existing coordination work.

Further, how clinicians are to fulfil their expanded roles within existing work practices is left unaddressed. The adequacy of hospital professionals in clinical positions as coordinators responsible for patient pathways depends on both contextual and personal factors. In some hospital units, coordinators may have access to appropriate system support such as suitable information systems, multidisciplinary resources and organized teamwork, while others lack all such resources.

The studied policy documents use a ‘pathway rhetoric’ that is captivating. However, these concepts are established for disease-specific clinical pathways. Equating these different types of pathways may obscure the particular challenges inherent to creating coherent care pathways for patients with long-term needs, multimorbidity, low predictability of needs, or with involvement of several professionals, service units and institutions.

Finally, we argue that it is demanding to question the framing of the ‘problem’ and further to create opportunities for discussing alternative understandings, when personally responsible coordinators as the instrument for achieving the goal of coherent care pathways is obligated by law and seemingly solves an obvious challenge.

## Additional File

The additional file for this article can be found as follows:

- ijic-18-3-3617-s1.pdf
  **List of analysed documents** The included documents and where to find them on the Internet. DOI: https://doi.org/10.5334/ijic.3617.s1
