# Supplementary material for: Policies Make Coherent Care Pathways a Personal Responsibility for Clinicians: A Discourse Analysis of Policy Documents about Coordinators in Hospitals
Source: Int J Integr Care. 2018 Jul 10;18(3):5. doi: 10.5334/ijic.3617 (PMC6078125; doi:10.5334/ijic.3617)
Supplement: Supplementary file 1 [file ijic-18-3-3617-s1.pdf]

Policies make coherent care pathways a personal responsibility for clinicians: A discourse analysis of policy documents about coordinators in hospitals

Audhild Høyem, Deede Gammon, Gro Rosvold Berntsen and Aslak Steinsbekk

List of analysed documents

Accessed 6 July 2018

**Document number, document title and which parts of the documents are analysed**	**URL**
1. Specialized Health Services Act [12]. §§ 2-2, 2-5a, b and c	https://lovdata.no
2. Regulations to the Specialized Health Services Act and the Health and Care Services Act concerning rehabilitation, individual plan and patient care coordinator [28]	https://lovdata.no
3. Directive to the Specialized Health Services Act [26]. P. 23-27	https://helsedirektoratet.no/publikasjoner/spesialisthelsetjenesteloven-med-kommentarer
4. Law proposition to the Parliament, Prop. 125 L. Amendments to the Specialized Health Care Act. [25] Chapters 1-8, p. 5-38 and 10, p. 43-46	https://www.regjeringen.no/no/dokumenter/prop.-125-l-20142015/id2412753/
5. Guidelines for patient care coordinator [29]. Chapter 13, p. 82-93	https://helsedirektoratet.no/Retningslinjer/Rehabilitering,%20habilitering,%20individuell%20plan%20og%20koordinator.pdf.
6. Guidelines for contact physician [30]. Chapters 1-8, p. 1-33	https://helsedirektoratet.no/retningslinjer/veileder-for-kontaktlege-i-spesialisthelsetjenesten.
7. The Coordination Reform. Proper treatment – at the right place and right time. Report No. 47 (2008–2009) to the Storting. [11]. Chapters 1-5, p.11-53 and 10, p. 111-114	https://www.regjeringen.no/no/dokumenter/stmeld-nr-47-2008-2009-/id567201/
8. NOU 2005: 3. From piecemeal to whole - an integrated health service [31]. Chapters 1, 2, p. 11-21, 4, p. 40-48, 6 and 7, p. 67-87	https://www.regjeringen.no/no/dokumenter/nou-2005-03/id152579/.
9. NOU 1997: 2. The patient first! Leadership and organization in hospitals [32]. Chapters 2, p.15-16 and 8, p.92-108	https://www.regjeringen.no/no/dokumenter/nou-1997-2/id140689/sec1
10. Meld.St.11 (2015-2016) National health- and hospital plan 2016-2019. [33]. Chapter 7.3, p. 57-58	https://www.regjeringen.no/no/dokumenter/meld.-st.-11-20152016/id2462047/
